# Early postoperative liver function parameters as predictors of post-hepatectomy liver failure

**DOI:** 10.3389/fsurg.2025.1669938

**Published:** 2025-10-21

**Authors:** Schaima Abdelhadi, Mohamad El-Ahmar, Katharina Vedder, Mahmoud Halawa, Vanessa Orth, Maike Hermann, Meik Mönnichs, Georgi Vassilev, Christoph Reissfelder, Flavius Sandra-Petrescu

**Affiliations:** 1 Department of Surgery, Universitätsmedizin Mannheim, Medical Faculty Mannheim, Heidelberg University, Mannheim, Germany; 2 DKFZ Hector Cancer Institute at the University Medical Center Mannheim, Mannheim, Germany

**Keywords:** PHLF, liver failure, early prediction, bilirubin, transaminase, liver resection, INR, ISGLS

## Abstract

**Background:**

Post-hepatectomy liver failure (PHLF) is a serious complication after liver resection and is associated with increased morbidity and mortality. The current International Study Group of Liver Surgery (ISGLS) definition relies on laboratory values from postoperative day (POD) 5 onwards, which may potentially delay diagnosis and intervention. This study aimed to evaluate whether early postoperative liver function parameters can predict the development of PHLF.

**Methods:**

All patients who underwent elective liver resection between April 2019 and May 2023 were included in the study. Exclusion criteria were emergency or multivisceral resections and incomplete laboratory data. Bilirubin, international normalized ratio (INR), aspartate aminotransferase (AST), and alanine aminotransferase (ALT) were measured on POD 1, 3, and 5. Univariate and multivariate logistic regression analyses were performed to identify independent predictors of PHLF. Receiver operating characteristic (ROC) analysis was performed, and optimal cutoffs on POD3 were determined using the Youden index.

**Results:**

Out of 445 included patients, 38 (8.5%) developed PHLF. Bilirubin, INR, AST, and ALT levels were significantly higher in patients with PHLF from POD 1 onwards. On POD 3, bilirubin ≥1.8 mg/dl (AUC 0.79; sensitivity 93.3%, specificity 62.4%), INR ≥ 1.18 (AUC 0.83; sensitivity 80.6%, specificity 68.8%), AST ≥ 179 U/L (AUC 0.75; sensitivity 68.4%, specificity 74.9%), and ALT ≥ 258 U/L (AUC 0.70; sensitivity 68.8%, specificity 69.8%) demonstrated predictive value. In multivariate analysis, major hepatectomy, bilirubin on POD 3, INR on POD 3, and persistently elevated AST and ALT were confirmed as independent predictors of PHLF.

**Conclusion:**

Bilirubin and INR on POD 3 were the strongest independent predictors of PHLF. Elevated AST and ALT on POD 3 were also valuable prognostic indicators. Relying solely on ISGLS criteria from POD 5 onward may therefore delay diagnosis and intervention. Persistently elevated transaminases should be acknowledged as early indicators of liver dysfunction and considered in future revisions of PHLF definitions.

## 1 Introduction

Liver resection is a cornerstone in the treatment of primary and secondary hepatic malignancies. Despite advances in surgical techniques, perioperative care, and patient selection, post-hepatectomy liver failure (PHLF) remains one of the most feared complications (1–3). Reported incidence rates vary widely, ranging from 5% to over 30%, depending on the extent of resection, underlying liver function, and patient characteristics (2, 4). PHLF is associated with high morbidity, prolonged hospitalization, and mortality rates exceeding 50% in severe cases (2, 3). Given these consequences, early recognition of patients at risk is essential to optimize postoperative management and to initiate timely interventions.

To improve consistency in reporting across studies, the International Study Group of Liver Surgery (ISGLS) established a standardized definition and grading system for PHLF (1). According to this definition, PHLF is diagnosed based on elevated bilirubin and international normalized ratio (INR) values that occur on or after postoperative day (POD) 5 (1). While this framework has become the clinical standard, it is inherently time-dependent and may delay diagnosis and, consequently, the initiation of therapeutic measures (5–7).

In contrast, earlier attempts to predict postoperative liver dysfunction have relied on different biochemical criteria. For example, the albumin–bilirubin (ALBI) score has been applied to evaluate hepatic reserve (7). However, these models also focus on parameters measured relatively late in the postoperative course. Thus, their utility for early detection remains limited.

Early postoperative changes in liver function tests, such as aspartate aminotransferase (AST), alanine aminotransferase (ALT), bilirubin, and INR, are commonly observed following hepatic resection (8, 9), transaminase elevations have usually been interpreted as nonspecific markers of surgical stress or ischemia–reperfusion injury, and are not incorporated into widely used definitions of PHLF (5). Nevertheless, recent studies suggest that persistent or pronounced alterations in these parameters during the first 72 h after surgery may be indicative of impaired functional recovery and could serve as early warning signs of impending liver failure (10–12).

Despite these insights, the clinical relevance of routine early postoperative liver function parameters remains uncertain, and no consensus exists on their predictive value for PHLF. Therefore, the present study aimed to investigate whether early postoperative changes in bilirubin, INR, AST, and ALT are associated with the subsequent development of PHLF. Establishing reliable early predictors could enable clinicians to identify high-risk patients before POD 5, allowing closer monitoring, timely interventions, and ultimately improved outcomes after liver resection.

## 2 Methods

### 2.1 Study design and patient cohort

All consecutive patients who underwent liver surgery between April 2019 and April 2023 were identified from a prospectively maintained institutional database at the Department of Surgery, University Hospital Mannheim, Heidelberg University. Patients were eligible for inclusion if they were aged 18 years or older and underwent elective liver resection. Exclusion criteria were emergency liver resections and multivisceral resections. Additionally, we excluded patients with incomplete postoperative laboratory data. This cohort study was conducted in accordance with the STROCSS guidelines and was approved by the ethics committee at Heidelberg University (2024-839) (13). The study was retrospectively registered in the German Clinical Trials Register (DRKS00037463).

### 2.2 Definitions and data acquisition

We extracted demographic, clinical, intraoperative, and postoperative data from institutional electronic medical records. Laboratory parameters [AST, ALT, bilirubin, INR, albumin, platelets, alkaline phosphatase (AP), and gamma-glutamyltransferase (GGT)] were recorded preoperatively and on POD 1, 3, and 5. Liver resections were classified according to the Brisbane 2000 terminology (14). Anatomic Liver resections were defined in line with Couinaud's portal segmentation system as the complete removal of one or more portal territories along with the corresponding hepatic parenchyma (14).

Postoperative complications were classified according to the Clavien–Dindo classification (15). Liver-specific complications were defined and reported by the criteria established by the International Study Group of Liver Surgery (ISGLS) (1, 16). Patients with Child B cirrhosis were considered for resection only in carefully selected cases with preserved liver function (Child B7) and in the absence of clinically significant portal hypertension. Previous hepatic resections, systemic treatments, and locoregional therapies were assessed for all patients, independent of the underlying diagnosis, and were therefore not limited to primary liver malignancy. The primary endpoint was the occurrence of PHLF.

### 2.3 Standardization of perioperative care

All patients received standardized pre-, intra-, and postoperative care based on institutional protocols implemented within a structured multidisciplinary framework. In oncologic cases, surgical indications were discussed preoperatively in multidisciplinary tumor board meetings. All procedures were performed by experienced hepatobiliary surgeons.

### 2.4 Statistical analysis

Statistical analyses were performed using JAMOVI software, version 2.2.2 (Sydney, Australia). Continuous variables were tested for distribution and are presented as the mean ± standard deviation (SD) if normally distributed or as the median with interquartile range (IQR) if skewed. Categorical variables are given as absolute numbers and percentages. Between-group comparisons were conducted using the *χ*^2^ test or Fisher's exact test for categorical variables and the Mann–Whitney U test or Student's t test, as appropriate, for continuous variables.

Receiver operating characteristic (ROC) curve analysis was performed to evaluate the predictive accuracy of early postoperative laboratory parameters (bilirubin, INR, AST, ALT) for the development of PHLF. Optimal cut-off values on POD3 were determined using the Youden index, which maximizes the sum of sensitivity and specificity. Diagnostic performance was reported as the area under the curve (AUC) with 95% confidence intervals, together with corresponding sensitivity and specificity values.

Logistic regression analysis was used to identify predictors of PHLF. Variables significant in univariate analysis were entered into multivariable models. We also specified a parsimonious model that included clinically justified covariates: extent of resection (major vs. minor) and POD3 bilirubin, INR, AST, and ALT. Statistical significance was defined as *p* < 0.05.

## 3 Results

**Figure 1:**
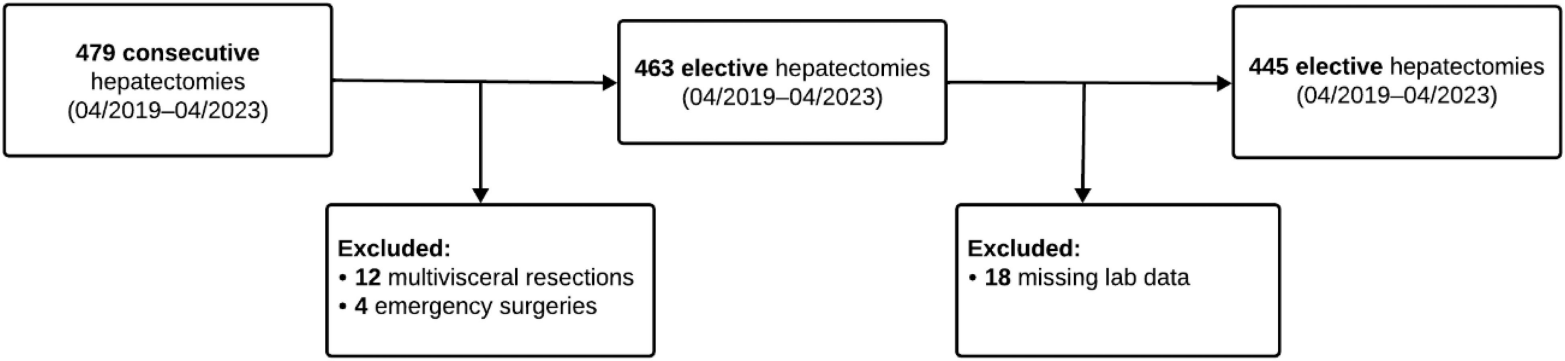
Patient flow chart.

A total of 479 consecutive liver resections were performed at our institution between April 2019 and April 2023. After excluding 4 emergency resections, 12 multivisceral resections, and 18 cases with incomplete perioperative laboratory data, 445 patients who underwent elective liver resections were included in the final analysis (Figure 1). Among the study population, 38 patients (8.5%) developed PHLF, while 407 patients (91.5%) did not.

### 3.1 Patient baseline and operative characteristics

Patient demographics and operative characteristics are summarized in Table 1. Baseline characteristics, including age, sex, body mass index (BMI), ASA classification, comorbidities, and liver cirrhosis status, were well balanced between groups. No significant differences were observed regarding the etiology of liver disease or previous treatments, including prior hepatic resection, locoregional therapy, or systemic therapy.

**Table 1:** Baseline characteristics and intraoperative variables of the study cohort.

Variables	Total (*n* = 445)	Non-PHLF (*n* = 407)	PHLF (*n* = 38)	*p*-value
Age, years^a^	66 (56–74)	66 (56–74)	67 (57–75)	0.53
BMI, kg/m^2^^a^	26 (23–29)	26 (23–29)	26 (25–30)	0.32
Sex ratio, Male: Female	273:172	247:160	26:12	0.35
ASA^b^				0.29
I	20 (4)	20 (5)	0 (0)	
II	226 (51)	210 (52)	16 (42)	
III	193 (43)	171 (42)	22 (58)	
IV	6 (1)	6 (1)	0 (0)	
Cardiovascular comorbidities^b^	262 (59)	239 (59)	23 (61)	0.83
Diabetes mellitus^b^	105 (24)	94 (23)	11 (29)	0.42
Pulmonary comorbidities^b^	69 (16)	61 (15)	8 (21)	0.32
Liver cirrhosis^b^	55 (13)	49 (12)	6 (16)	0.22
Child A	50 (11)	45 (11)	5 (13)	
Child B	5 (1)	4 (1)	1 (3)	
Etiology of cirrhosis^b^				0.22
Alcohol	29 (7)	27 (7)	2 (5)	
Viral	19 (4)	15 (4)	4 (11)	
Hepatitis B	12 (3)	9 (2)	3 (8)	
Hepatitis C	7 (2)	6 (1)	1 (3)	
MASLD	7 (2)	7 (2)	0 (0)	
Diagnosis				**<0**.**01**
PHB Malignancy	161 (36)	137 (34)	24 (63)	
HCC	98 (22)	84 (21)	14 (39)	
CCC	58 (13)	48 (12)	10 (26)	
GBC	5 (1)	5 (1)	0 (0)	
Metastatic disease	217 (49)	208 (51)	9 (24)	
Benign	67 (15)	62 (15)	5 (13)	
Previous treatment^b^				
Previous hepatic resection	99 (22)	89 (22)	10 (27)	0.53
Previous locoregional therapy	19 (4)	19 (5)	0 (0)	0.17
Previous systemic treatment	22 (5)	19 (5)	3 (8)	0.19
Surgical approach^b^				
Laparoscopic	271 (61)	255 (63)	16 (42)	**<0**.**01**
Robotic	32 (7)	31 (8)	1 (3)	
Open	142 (32)	121 (30)	21 (55)	
Surgical procedure^b^				
Non-anatomic resections	147 (33)	143 (35)	4 (11)	**<0**.**01**
Right (extended) hepatectomy	82 (18)	63 (15)	19 (50)	
Left (extended) hepatectomy	41 (9)	38 (9)	3 (8)	
Left lateral sectionectomy	36 (8)	36 (9)	0 (0)	
Right posterior sectionectomy	17 (4)	15 (4)	2 (5)	
Right anterior sectionectomy	3 (1)	3 (1)	0 (0)	
Other Mono- or Bisegmentectomy	119 (27)	109 (27)	10 (26)	
Operative time, min^a^	243 (162–345)	236 (151–325)	339 (235–414)	**<0**.**01**
Pringle maneuver^b^	196 (44)	177 (43)	19 (50)	0.29
Duration, min^a^	30 (17–56)	30 (17–54)	27 (17–48)	0.95
Blood loss, ml^b^	500 (150–1,300)	450 (200–1,200)	1,200 (700–2,850)	**<0**.**01**
Intraoperative transfusion^b^				
pRBC	113 (25)	89 (22)	24 (63)	**<0**.**01**
FFP	157 (35)	131 (32)	26 (68)	**<0**.**01**

BMI, body mass index; ASA, American Society of Anesthesiologists; PHB, primary hepatobiliary malignancy; HCC, hepatocellular carcinoma; CCC, cholangiocarcinoma; GBC, gallbladder cancer; pRBC, packed red blood cells; FFP, fresh frozen plasma.

*p*-values in bold indicate statistical significance (*p* < 0.05).

a Values are median (interquartile range).

b Values are presented as absolute numbers (percentages).

Major hepatectomies were more frequently performed in the PHLF group than in non-PHLF patients (58% vs. 25%, *p* < 0.001). The PHLF group also had significantly longer operative times (median 339 vs. 236 min, *p* < 0.001), higher intraoperative blood loss (median 1,200 vs. 450 mL, *p* < 0.001), and more frequent need for intraoperative transfusions (pRBC: 63% vs. 22%, *p* < 0.001; FFP: 68% vs. 32%, *p* < 0.001). Open resections were also more common in the PHLF group (55% vs. 30%, *p* < 0.001).

### 3.2 Postoperative liver function parameters

**Figure 2:**
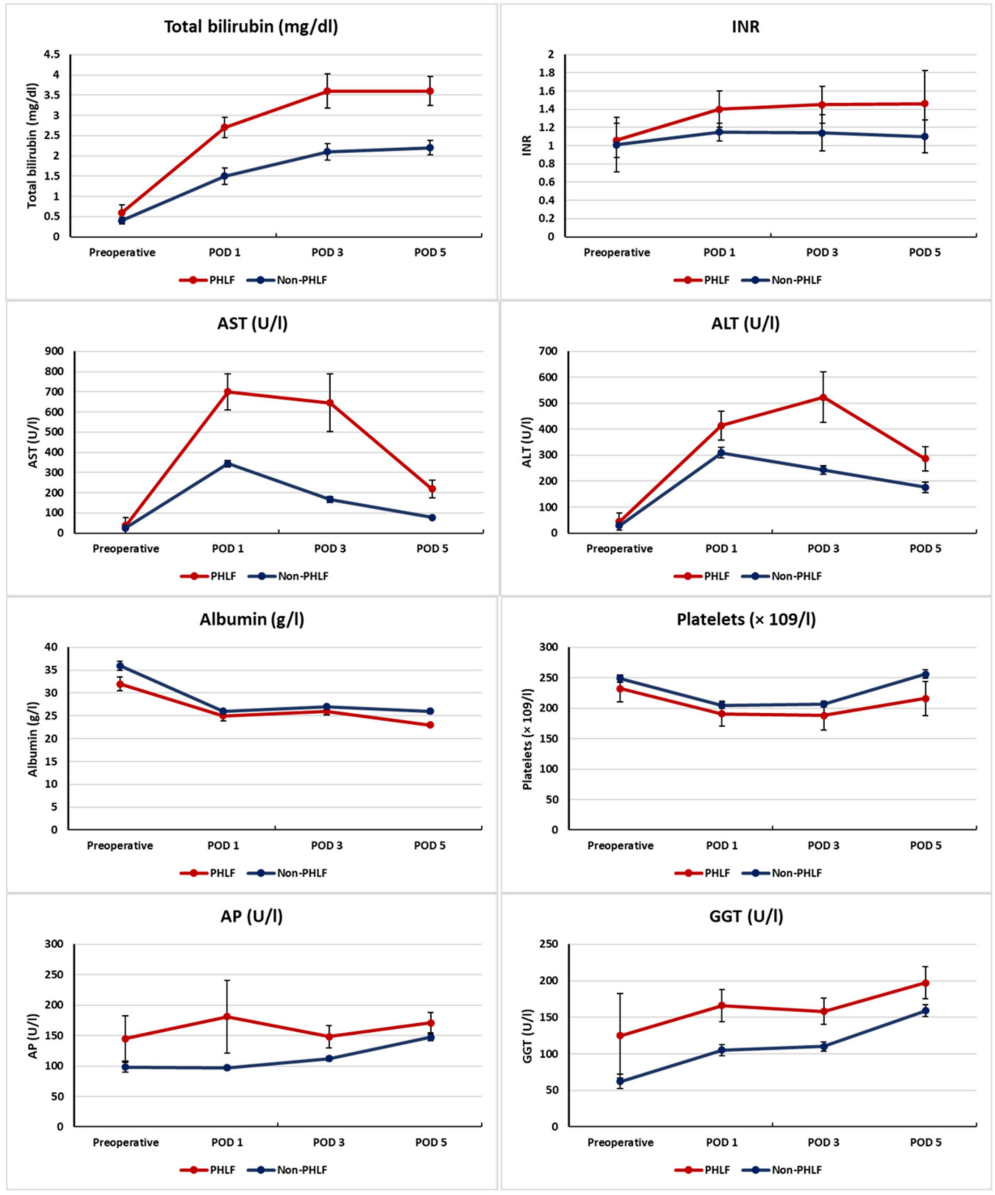
Postoperative course of liver function parameters in patients with and without PHLF. Median values (IQR) of bilirubin, INR, AST, ALT, albumin, and platelet count from the preoperative baseline to POD5 are shown. Red lines indicate patients with PHLF, blue lines those without PHLF.

Postoperative liver function parameters are summarized in Table 2; Figure 2.

**Table 2:** Perioperative liver function tests stratified by PHLF Status.

Variables	Total (*n* = 445)	Non-PHLF (*n* = 407)	PHLF (*n* = 38)	*p*-value
Albumin (g/L)				
Preoperative	35.6 (30.7–39.1)	36 (30.9–39.1)	32 (30.7–36.5)	0.47
POD 1	27.0 (23.6–30.1)	26 (22.2–29.2)	25 (22.2–29.2)	**0**.**02**
POD 3	26.5 (23.5–29.2)	27 (23.2–29.2)	26 (23.6–28.3)	**<0**.**001**
POD5	26.0 (22.0–30.0)	26 (24–30.1)	23 (20.2–27.6)	**0**.**06**
Bilirubin (mg/dl)				
Preoperative	0.48 (0.32–0.72)	1.1 (0.44–1.42)	1.2 (0.72–1.84)	0.20
POD 1	0.99 (0.66–1.54)	1.5 (1.2–2.08)	2.7 (1.59–4.31)	**0**.**011**
POD 3	1.71 (1.20–2.60)	2.1 (1.54–2.46)	3.6 (2.46–4.38)	**<0**.**001**
POD 5	1.64 (1.20–3.46)	2.2 (1.59–2.76)	3.6 (2.46–4.38)	**<0**.**001**
INR				
Preoperative	1.02 (0.97–1.07)	1.01 (0.97–1.06)	1.06 (1.01–1.13)	**0**.**01**
POD 1	1.14 (1.08–1.21)	1.14 (1.08–1.19)	1.27 (1.19–1.36)	**<0**.**001**
POD 3	1.13 (1.06–1.25)	1.12 (1.05–1.20)	1.35 (1.20–1.42)	**<0**.**001**
POD 5	1.10 (1.04–1.20)	1.08 (1.03–1.15)	1.30 (1.15–1.42)	**<0**.**001**
Platelets (×10^9 ^/L)				
Preoperative	249 (193–319)	249 (193–330)	232 (152–319)	0.91
POD 1	183 (142–263)	182 (142–226)	135 (107–186)	0.37
POD 3	180 (138–245)	182 (142–245)	135 (107–245)	0.28
POD 5	219 (168–301)	221 (174–301)	186 (125–244)	0.07
AP (U/L)				
Preoperative	106 (80–164)	106 (80–157)	147 (98–305)	**0**.**001**
POD 1	124 (91–174)	124 (91–147)	171 (109–202)	**<0**.**001**
POD 3	93 (69–131)	93 (69–128)	178 (109–342)	**0**.**007**
POD 5	119 (83–208)	119 (83–185)	192 (131–244)	0.13
GGT (U/L)				
Preoperative	62 (26–136)	62 (26–147)	136 (62–305)	**<0**.**001**
POD 1	64 (43–109)	64 (43–136)	192 (109–270)	**0**.**01**
POD 3	75 (43–140)	75 (43–128)	283 (152–342)	**0**.**01**
POD 5	124 (96–208)	124 (96–208)	216 (125–244)	0.11
AST (U/L)				
Preoperative	26 (19–38)	26 (19–43)	36 (26–64)	**<0**.**001**
POD 1	241 (118–447)	232 (118–447)	619 (263–826)	**<0**.**001**
POD 3	103 (73–200)	99 (73–186)	283 (152–520)	**<0**.**001**
POD 5	59 (43–96)	56 (42–125)	131 (71–186)	**<0**.**001**
ALT (U/L)				
Preoperative	28 (19–65)	26 (19–43)	43 (26–98)	**0**.**006**
POD 1	205 (146–286)	188 (142–263)	342 (192–520)	**0**.**05**
POD 3	167 (135–245)	152 (109–245)	270 (174–420)	**<0**.**001**
POD 5	94 (59–131)	83 (56–131)	192 (125–301)	**0**.**04**

INR, international normalized ratio; AP, alkaline phosphatase; GGT, gamma-glutamyl-transferase; AST, aspartate aminotransferase; ALT, alanine aminotransferase.

*p*-values in bold indicate statistical significance (*p* < 0.05).

Values are median (interquartile range).

Patients with PHLF showed consistently higher bilirubin levels throughout the early postoperative period. Median bilirubin was significantly elevated on POD 1 [2.7 [1.6–3.4] vs. 1.5 [1.2–2.1] mg/dL, *p* = 0.011], POD 3 [3.6 [2.6–4.4] vs. 2.1 [1.5–2.5] mg/dL, *p* < 0.001], and POD 5 [3.6 [2.5–4.4] vs. 2.2 [1.6–2.8] mg/dL, *p* < 0.001].

INR values were also significantly increased in the PHLF group at all postoperative time points, with differences already evident on POD 1 [1.27 [1.19–1.36] vs. 1.14 [1.08–1.19], *p* < 0.001], and remaining significant on POD 3 [1.35 [1.20–1.42] vs. 1.12 [1.05–1.20], *p* < 0.001] and POD 5 [1.30 [1.15–1.42] vs. 1.08 [1.03–1.15], *p* < 0.001].

Serum transaminases were markedly elevated in the PHLF group. AST levels were higher on POD 1 [619 (263–826) vs. 232 (118–447) U/L, *p* < 0.001], POD 3 [283 (152–520) vs. 99 (73–186) U/L, *p* < 0.001], and POD 5 [131 (71–186) vs. 56 (42–125) U/L, *p* < 0.001]. ALT values showed a similar trend, with significantly elevated levels on POD 3 [270 (174–420) vs. 152 (109–245) U/L, *p* < 0.001] and POD 5 [192 (125–301) vs. 83 (56–131) U/L, *p* = 0.04].

In contrast, albumin levels were consistently lower in the PHLF group but did not reach statistical significance. Median values were 25 (22–29) vs. 26 (22–29) g/L on POD 1 (*p* = 0.02), 26 (23–28) vs. 27 (23–29) g/L on POD 3 (*p* < 0.001), and 23 (20–27) vs. 26 (24–30) g/L on POD 5 (*p* = 0.06).

Platelet counts were also lower in the PHLF group but without significant differences [POD 5: 186 (125–244) vs. 221 (174–301) × 10^9^ /L, *p* = 0.07].

### 3.3 Predictive factors for PHLF

To identify predictive factors for PHLF, univariate and multivariate logistic regression analyses were performed (Supplementary Tables S1, S3).

In univariate analysis, several intraoperative factors were associated with the development of PHLF, including the extent of resection (OR 4.17; 95% CI 2.12–8.37; *p* < 0.001), open surgical approach (OR 2.77; 95% CI 1.40–5.57; *p* = 0.004), longer operative time (OR 1.00; 95% CI 1.00–1.01; *p* < 0.001), blood loss (OR 1.00; 95% CI 1.00–1.00; *p* < 0.001), transfusion of pRBC (OR 6.11; 95% CI 3.07–12.58; *p* < 0.001), and transfusion of FFP (OR 4.56; 95% CI 2.28–9.65; *p* < 0.001).

Among the postoperative laboratory parameters, lower albumin levels on POD 1 (OR 0.96; 95% CI 0.92–1.00; *p* = 0.026), POD 3 (OR 0.90; 95% CI 0.84–0.96; *p* = 0.001), and POD 5 (OR 0.92; 95% CI 0.86–0.98; *p* = 0.011) were significantly associated with PHLF. Elevated bilirubin on POD 3 (OR 1.45; 95% CI 1.18–1.83; *p* = 0.001) and POD 5 (OR 1.43; 95% CI 1.15–1.85; *p* = 0.003) as well as INR on POD 3 (OR 2.0 × 10^3^; 95% CI 178.67–4.0 × 10^4^; *p* < 0.001) and POD 5 (OR 14 × 10^3^; 95% CI 361.44–1.1 × 10^6^; *p* < 0.001) showed strong associations. AST was predictive on POD 1, 3, and 5, and ALT on POD 3 (all *p* < 0.01).

In the multivariate analysis (Table 3), extent of resection (OR 2.75; 95% CI 1.25–6.54; *p* = 0.013), bilirubin on POD 3 (OR 1.42; 95% CI 1.14–1.83; *p* = 0.003) and POD 5 (OR 1.38; 95% CI 1.08–1.83; *p* = 0.015), INR on POD 3 (OR 24.93; 95% CI 0.01–9.0 × 10^4^; *p* = 0.002) and POD 5 (OR 367.38; 95% CI 0.57–1.4 × 10^6^; *p* = 0.001), as well as AST on POD 1–5 and ALT on POD 3 remained significantly associated with PHLF.

**Table 3:** Multivariate logistic regression analysis of predictive factors for PHLF.

Variables		Multivariate	*p*-value
	OR	95%CI	
Extent of resection (Major vs. Minor)	2.75	1.25–6.54	**0**.**013**
Bilirubin (mg/dl)			
POD 3	1.42	1.14–1.83	**0**.**003**
POD 5	1.38	1.08–1.83	**0**.**015**
INR			
POD 3	24.93	0.01–9 × 10^4^	**0**.**002**
POD 5	367.38	0.57–1.4 × 10^6^	**0**.**001**
AST (U/L)			
POD 1	1.00	0.999–1.001	**0**.**002**
POD 3	1.00	0.999–1.003	**0**.**005**
POD 5	1.01	0.999–1.009	**0**.**040**
ALT (U/L)			
POD 3	1.00	0.999–1.009	**0**.**005**

INR, international normalized ratio; AST, aspartate aminotransferase; ALT, alanine aminotransferase.

*p*-values in bold indicate statistical significance (*p* < 0.05).

To account for the limited number of events, a parsimonious model was additionally tested, including the extent of resection and POD3 bilirubin, INR, AST, and ALT (Supplementary Table S2). In this model, bilirubin (OR 1.382; 95% CI 1.082–1.781; *p* = 0.012), INR (OR 2.0 × 10^3^; 95% CI 5.4–1.1 × 10^4^; *p* = 0.005), and AST (OR 1.021; 95% CI 1.001–1.041; *p* = 0.032) remained independent predictors of PHLF. ALT did not reach statistical significance (OR 1.001; 95% CI 1.001–1.003; *p* = 0.061).

### 3.4 Threshold analysis

**Figure 3:**
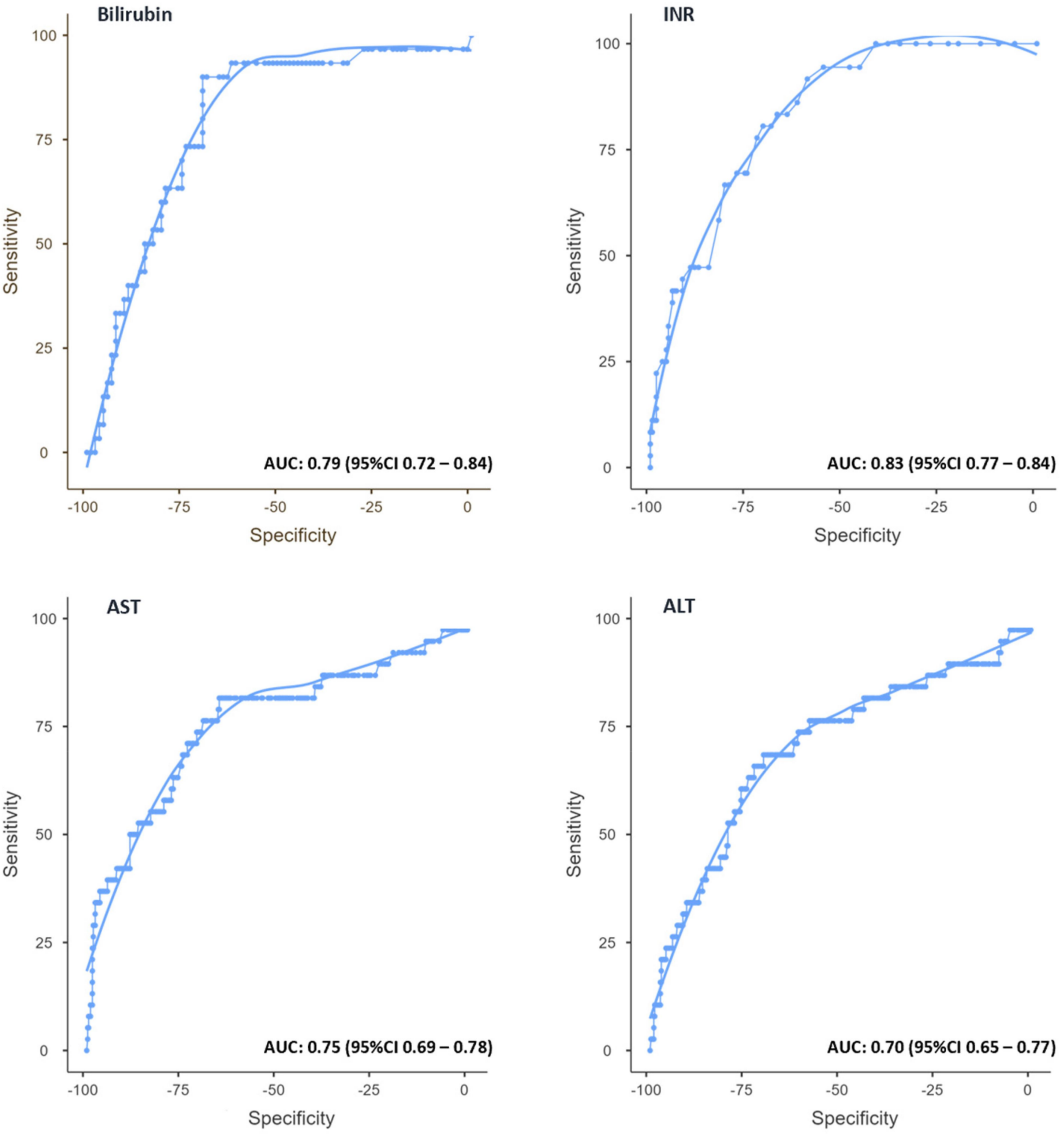
Receiver operating characteristic (ROC) curves for bilirubin, INR, AST, and ALT on POD 3. ROC curves with optimal cutoff values, sensitivities, and specificities for predicting PHLF. Area under the curve (AUC) values are indicated for each parameter.

In the next step, the predictive validity of early postoperative liver function parameters for PHLF was assessed through ROC analysis (Figure 3). The optimal cut-off value for bilirubin on POD 3 was determined as 1.8 mg/dl, yielding a sensitivity of 93.3% and a specificity of 62.4% (AUC: 0.79; 95% CI 0.72–0.84). For INR, the best threshold was 1.18, with a sensitivity of 80.6% and specificity of 68.8% (AUC: 0.83; 95% CI 0.77–0.84).

Regarding transaminases, AST showed an optimal cutoff value of 179 U/L, predicting PHLF with a sensitivity of 68.4% and a specificity of 74.9% (AUC: 0.75; 95% CI 0.69–0.78). For ALT, the best threshold was 258 U/L, corresponding to a sensitivity of 68.8% and specificity of 69.8% (AUC: 0.70; 95% CI 0.65–0.77).

## 4 Discussion

In our large single-center analysis, early postoperative changes in bilirubin, INR, AST, and ALT emerged as significant independent predictors of PHLF. These parameters already differed significantly between patients with and without PHLF on POD1, with predictive accuracy peaking on POD3, suggesting that clinically meaningful warning signs can be identified earlier than currently recognized.

Importantly, AST and ALT levels were also significantly elevated in patients with PHLF and remained independent predictors even after adjustment for confounders. Traditionally, postoperative transaminase elevations have been considered nonspecific markers of hepatocellular injury or surgical stress, particularly in the early postoperative phase (5) and have consequently not been incorporated into commonly used definitions or predictive models of PHLF, such as the “50–50” criteria, which rely exclusively on bilirubin and INR levels on POD 5 (6), or the ALBI score, which is based on albumin and bilirubin concentrations (7). Similarly, the ISGLS definition of PHLF does not account for transaminase dynamics in its diagnostic criteria (1).

However, our findings challenge this convention by demonstrating that AST and ALT elevations as early as POD 1 are independently associated with PHLF. Notably, values measured on POD 3 showed the highest predictive accuracy in our cohort. This suggests that failure of transaminase levels to decline, or their continued rise in the early postoperative period, may reflect ongoing hepatocellular damage or impaired recovery of liver function.

When comparing the predictive value of AST and ALT, AST appeared to be the more reliable marker in the early postoperative phase. A likely explanation is its predominantly mitochondrial localization. While ALT is primarily found in the cellular cytoplasm, AST is present in both the cytosol (approximately 20% of total activity) and the mitochondria (approximately 80% of total activity) of hepatocytes (8). This mitochondrial component might reflect more extensive or prolonged hepatocellular damage, particularly in the context of ischemia-reperfusion injury (8, 9, 17–21). Persistent AST elevation beyond POD 1 could therefore not only reflect the extent of liver cell injury, but also give indirect insight into the functional reserve or metabolic stability of the remnant liver. ALT remains a valid parameter, but may be less specific in this context.

Our findings are consistent with previous studies that underline the relevance of early postoperative changes in liver function parameters. In a recent study, bilirubin, INR, AST, and ALT levels on POD 1 were all associated with the development of PHLF (10). Interestingly, AST levels greater than 260 U/L were identified as an independent predictor in multivariate analysis, whereas ALT did not remain significant (10). This discrepancy may be attributed to differences in patient selection and surgical techniques. Our cohort included a wider range of resections and a higher median patient age, which may have influenced the postoperative risk profile and inflammatory response.

Another study highlighted the relevance of POD 1 AST levels in patients undergoing major liver resection for colorectal liver metastases, proposing an AST cut-off of 798 U/L for predicting 90-day mortality (11). Although our identified thresholds were lower, the consistent early rise in AST, along with its independent predictive value, supports the utility of transaminases as early markers of hepatic dysfunction. A further study analyzed the kinetics of postoperative transaminase levels in patients with HBV-related HCC and PHLF (12). In this study, a delayed peak of ALT beyond postoperative day 3 (PDE-ALT) was significantly associated with increased 30-day mortality, which suggests that PDE-ALT may serve as an early predictor of lethal PHLF (12).

In contrast to our results, another study did not identify postoperative transaminase levels as independent predictors of morbidity following hepatectomy (5). However, in this study, the definition of postoperative morbidity included a wide range of complications such as pulmonary events, hemorrhage, and wound infections. Importantly, only 3% of the complications were classified as PHLF (5). This difference in outcome definition likely explains the divergent findings, as transaminase elevations are more specifically related to hepatocellular injury rather than to systemic complications unrelated to liver function.

Neither AP nor GGT showed a significant association with PHLF in our analysis. This may be explained by their lower sensitivity to acute hepatocellular injury and their limited dynamic response in the early postoperative phase (22). While both enzymes primarily reflect cholestasis and biliary tract integrity (22), their utility as early predictors of hepatic insufficiency appears to be limited in this context.

Interestingly, although lower albumin levels were significantly associated with PHLF in univariate analysis, they did not retain predictive power in the multivariate model, likely reflecting the multifactorial nature of albumin metabolism and its limited utility as a predictive marker in the early postoperative period (23).

In line with previous studies, intraoperative factors such as major hepatectomy, longer operative time, increased blood loss, and the need for transfusions were also associated with PHLF in univariate analysis. Of these, only major hepatectomy remained an independent risk factor. This highlights the physiological burden of extensive parenchymal loss and the importance of preserving an adequate future liver remnant (2, 4).

The strengths of our study include a large and well-characterized single-center cohort, standardized postoperative laboratory monitoring, and the use of established ISGLS criteria for outcome definition. However, certain limitations must be acknowledged. First, it is a retrospective, prognostic study with a potential selection and reporting bias. Second, the identified cut-off values in our study were not tested in a separate cohort to verify the optimal cut-off values. Lastly, our results were obtained from a single-center study, which may not apply to other populations with different etiologies of liver disease.

Taken together, our findings demonstrate the importance of routine early postoperative monitoring of bilirubin, INR, AST, and ALT for the timely identification of patients at risk for PHLF. Rather than awaiting fulfillment of ISGLS criteria on or after POD 5, clinicians should interpret persistently elevated or rising transaminase and bilirubin levels as early warning signs. This is especially relevant following major resections, where regenerative capacity is limited and early therapeutic decisions may be critical.

From a clinical perspective, such developments should trigger close monitoring, ideally in an intermediate or intensive care unit, as well as a structured approach to fluid management and the temporary discontinuation of potentially hepatotoxic medications, such as paracetamol, statins, antidepressants, or non-steroidal anti-inflammatory drugs (24).

Given accumulating evidence supporting the predictive role of transaminases and the practical utility of bilirubin and INR before POD 5, it may be time to reconsider and refine the current ISGLS definition of PHLF. Future prospective studies should focus not only on validating cut-off values, but also on determining whether biochemically guided early rescue strategies can meaningfully alter the course of PHLF.

## Data Availability

The raw data supporting the conclusions of this article will be made available by the authors, without undue reservation.
