# Supplementary material for: Early postoperative liver function parameters as predictors of post-hepatectomy liver failure
Source: Front Surg. 2025 Oct 21;12:1669938. doi: 10.3389/fsurg.2025.1669938 (PMC12583028; doi:10.3389/fsurg.2025.1669938)
Supplement: Supplementary file 1 [file Table1.docx]

**Supplementary Material**

**Supplementary 1: Univariate and Multivariate Analysis of Predictive Factors for PHLF**

|  | |  | | | **Univariate** | | | |  | |  | | **Multivariate** | | | |
| --- | --- | --- | --- | --- | --- | --- | --- | --- | --- | --- | --- | --- | --- | --- | --- | --- |
| **Preoperative variables** | | | **OR** | | | **95%CI** | | | **P** |  | | | **OR** | | **95%CI** | **P** |
| **Age, years** | | | 1.01 | | | 0.98 – 1.04 | | | 0.534 | | | |  | |  |  |
| **BMI, kg/m^2^** | | | 1.03 | | | 0.97 – 1.10 | | | 0.322 | | | |  | |  |  |
| **Female vs. Male sex** | | | 0.71 | | | 0.34 – 1.42 | | | 0.351 | | | |  | |  |  |
| **ASA** | | |  | | |  | | |  | | | |  | |  |  |
| ASA II vs. ASA I | | | 1.44 | | | 0.27 – 26.62 | | | 0.733 | | | |  | |  |  |
| ASA III vs. ASA I | | | 2.56 | | | 0.49 – 47.01 | | | 0.371 | | | |  | |  |  |
| ASA IV vs. ASA I | | | 2.86 | | | 0.10 – 79.13 | | | 0.478 | | | |  | |  |  |
| **Cardiovascular comorbidities** (Yes vs. No) | | | 1.08 | | | 0.55 – 2.17 | | | 0.829 | | | |  | |  |  |
| **Diabetes mellitus** (Yes vs. No) | | | 1.36 | | | 0.62 – 2.77 | | | 0.418 | | | |  | |  |  |
| **Pulmonary comorbidities** (Yes vs. No) | | | 1.51 | | | 0.62 – 3.31 | | | 0.326 | | | |  | |  |  |
| **Liver cirrhosis** (Child B vs. Child A) | | | 1.37 | | | 0.50 – 3.23 | | | 0.503 | | | |  | |  |  |
| **Etiology of cirrhosis** | | |  | | |  | | |  | | | |  | |  |  |
| Viral vs. Alcohol | | | 3.41 | | | 0.50 – 28.79 | | | 0.212 | | | |  | |  |  |
| MASLD vs. Alcohol | | | 3.13 | | | 0.13 – 41.43 | | | 0.394 | | | |  | |  |  |
| **Diagnosis** | | |  | | |  | | |  | | | |  | |  |  |
| PHB malignancy vs. benign | | | 5.08 | | | 1.44 – 32.26 | | | ***0.031*** | | | | 3.18 | | 0.28 – 81.72 | 0.393 |
| Metastatic disease vs. benign | | | 1.25 | | | 0.31 – 2.38 | | | 0.775 | | | |  | |  |  |
| **Previous treatment** | | |  | | |  | | |  | | | |  | |  |  |
| Previous hepatic resection (Yes vs. No) | | | 1.43 | | | 0.63 – 2.99 | | | 0.364 | | | |  | |  |  |
| Previous locoregional therapy (Yes vs. No) | | | < 0.01 | | | NA – 7x10^10^ | | | 0.986 | | | |  | |  |  |
| Previous systemic treatment (Yes vs. No) | | | 0.47 | | | 0.11 – 1.36 | | | 0.219 | | | |  | |  |  |
| **Intraoperative variables** | | |  | | |  | | |  | | | |  | |  |  |
| **Extent of resection** (Major vs. Minor) | | | 4.17 | | | 2.12–8.37 | | | ***<0.001*** | | | | 2.75 | | 1.25 – 6.54 | ***0.013*** |
| **Surgical approach** | | |  | | |  | | |  | | | |  | |  |  |
| Robotic vs. Laparoscopic | | | 0.51 | | | 0.03 – 2.66 | | | 0.526 | | | |  | |  |  |
| Open vs. Laparoscopic | | | 2.77 | | | 1.40 – 5.57 | | | ***0.004*** | | | | 2.09 | | 0.93 – 4.76 | 0.074 |
| **Operative time, min** | | | 1.00 | | | 1.00 –1.01 | | | ***<0.001*** | | | | 1.00 | | 1.00 – 1.00 | 0.598 |
| **Pringle maneuver** (Yes vs. No) | | | 0.70 | | | 0.15 – 5.22 | | | 0.681 | | | |  | |  |  |
| Duration, min | | | 1.00 | | | 0.98 – 1.01 | | | 0.950 | | | |  | |  |  |
| **Blood loss, ml** | | | 1.00 | | | 1.00 –1.01 | | ***<0.001*** | | | | | 1.00 | | 1.00 – 1.00 | 0.089 |
| **Intraoperative transfusion** | | |  | | |  | |  | | | | |  | |  |  |
| pRBC | | | 6.11 | | | 3.07–12.58 | | ***<0.001*** | | | | | 2.24 | | 0.75 – 7.29 | 0.161 |
| FFP | | | 4.56 | | | 2.28 – 9.65 | | ***<0.001*** | | | | | 0.97 | | 0.28 – 3.13 | 0.957 |
| **Laboratory** **variables** | | |  | | |  | |  | | | | |  | |  |  |
| **Albumin** (g/l) | | |  | | |  | |  | | | | |  | |  |  |
| Preoperative | | | 0.99 | | | 0.96 – 1.01 | | 0.383 | | | | |  | |  |  |
| POD 1 | | | 0.96 | | | 0.92 – 1.00 | | ***0.026*** | | | | | 1.00 | | 0.93 – 1.10 | 0.987 |
| POD 3 | | | 0.90 | | | 0.84 – 0.96 | | ***0.001*** | | | | | 0.99 | | 0.88 – 1.11 | 0.889 |
| POD5 | | | 0.92 | | | 0.86 – 0.98 | | ***0.011*** | | | | | 0.94 | | 0.81 – 1.04 | 0.397 |
| **Bilirubin (mg/dl)** | | |  |  | | | |  | | | | |  | |  |  |
| Preoperative | 1.10 | | | 0.91 – 1.27 | | | 0.599 | | | | |  | | |  |  |
| POD 1 | 1.03 | | | 0.97 – 1.10 | | | 0.193 | | | | |  | |  | |  |
| POD 3 | 1.45 | | | 1.18 – 1.83 | | | ***0.001*** | | | | | 1.42 | | 1.14 – 1.83 | | ***0.003*** |
| POD 5 | 1.43 | | | 1.15 – 1.85 | | | ***0.003*** | | | | | 1.38 | | 1.08 – 1.83 | | ***0.015*** |
| **INR** |  | | |  | | |  | | | | |  | |  | |  |
| Preoperative | 18.35 | | | 2.43 – 193.79 | | | 0.010 | | | | |  | |  | |  |
| POD 1 | 1.10 | | | 0.91 – 1.271 | | | 0.599 | | | | |  | |  | |  |
| POD 3 | 2x10^3^ | | | 178.6 – 4x10^4^ | | | ***<0.001*** | | | | | 24.93 | | 0.01 – 9x10^4^ | | ***0.002*** |
| POD 5 | 14x10^3^ | | | 361.4 –11x10^5^ | | | ***<0.001*** | | | | | 367.38 | | 0.57 – 1.4x10^6^ | | ***0.001*** |
| **Platelets (x10^9^/l)** |  | | |  | | |  | | | | |  | |  | |  |
| Preoperative | 1.003 | | | 0.996 – 1.001 | | | 0.915 | | | | |  | |  | |  |
| POD 1 | 1.001 | | | 0.996 – 1.001 | | | 0.378 | | | | |  | |  | |  |
| POD 3 | 1.001 | | | 0.996 – 1.001 | | | 0.281 | | | | |  | |  | |  |
| POD 5 | 1.001 | | | 0.996 – 1.001 | | | 0.079 | | | | |  | |  | |  |
| **AP (U/l)** |  | | |  | | |  | | | | |  | |  | |  |
| Preoperative | 1.011 | | | 1.001 – 1.014 | | | ***0.007*** | | | | | 1.001 | | 0.996 – 1.001 | | 0.728 |
| POD 1 | 1.012 | | | 1.001 – 1.013 | | | ***0.036*** | | | | | 1.001 | | 0.998 – 1.001 | | 0.255 |
| POD 3 | 1.011 | | | 1.001 – 1.014 | | | ***0.011*** | | | | | 1.001 | | 0.996 – 1.001 | | 0.080 |
| POD 5 | 1.0002 | | | 0.999 – 1.008 | | | 0.136 | | | | |  | |  | |  |
| **gGT (U/l)** |  | | |  | | |  | | | | |  | |  | |  |
| Preoperative | 1.012 | | | 1.001 – 1.014 | | | ***0.001*** | | | | | 1.001 | | 0.997 – 1.001 | | 0.404 |
| POD 1 | 1.011 | | | 1.001 – 1.012 | | | ***0.027*** | | | | | 1.001 | | 0.996 – 1.001 | | 0.306 |
| POD 3 | 1.011 | | | 1.001 – 1.014 | | | ***0.022*** | | | | | 1.001 | | 0.996 – 1.001 | | 0.124 |
| POD 5 | 1.0001 | | | 0.999 – 1.002 | | | 0.123 | | | | |  | |  | |  |
| **AST (U/l)** |  | | |  | | |  | | | | |  | |  | |  |
| Preoperative | 1.012 | | | 1.001 – 1.014 | | | ***0.022*** | | | | | 1.001 | | 0.999 – 1.009 | | 0.102 |
| POD 1 | 1.001 | | | 1.001 – 1.011 | | | ***<0.001*** | | | | | 1.001 | | 1.001 – 1.011 | | ***0.002*** |
| POD 3 | 1.001 | | | 1.001 – 1.011 | | | ***<0.001*** | | | | | 1.001 | | 1.001 – 1.011 | | ***0.005*** |
| POD 5 | 1.001 | | | 0.999 – 1.009 | | | ***<0.001*** | | | | | 1.001 | | 1.001 – 1.011 | | ***0.040*** |
| **ALT (U/l)** |  | | |  | | |  | | | | |  | |  | |  |
| Preoperative | 1.001 | | | 1.001 – 1.011 | | | ***0.027*** | | | | | 1.001 | | 0.999 – 1.001 | | 0.061 |
| POD 1 | 1.001 | | | 0.999 – 1.002 | | | 0.128 | | | | |  | |  | |  |
| POD 3 | 1.001 | | | 1.001 – 1.011 | | | | ***<0.001*** | | | | 1.001 | | 1.001 – 1.011 | | ***0.005*** |
| POD 5 | 1.001 | | | 0.999 – 1.001 | | | | 0.140 | | | |  | |  | |  |
| *p*-values in bold italics indicate statistical significance (*p* < 0.05). | | | | | | | | | | | | | | | | |
|  | | |  | | |  | |  | | | | |  | |  |  |

**Supplementary 2: Parsimonious model adjusted for extent of resection and POD3 laboratory parameters**

| **Variables** | **OR** | **95%CI** | ***p*-value** |
| --- | --- | --- | --- |
| **Extent of resection (major/minor)** | 0.912 | **0.317 – 3.02** | 0.561 |
| **Bilirubin on POD 3** | 1.382 | 1.082 – 1.781 | ***0.012*** |
| **INR on POD 3** | 2x10^3^ | 5.42 – 11x10^3^ | ***0.005*** |
| **AST on POD3** | 1.021 | 1.001 – 1.041 | ***0.032*** |
| **ALT on POD 3** | 1.001 | 1.001 – 1.003 | 0.061 |

*OR* odds ratio; *CI* confidence interval; *POD* postoperative day, *INR* international normalized ratio, *AST* aspartate aminotransferase, *ALT* alanine aminotransferase
*p*-values in bold italics indicate statistical significance (*p* < 0.05).
